# Effect of Intra-arterial Balloon Pumping for Refractory Septic Cardiomyopathy: A Case Series

**DOI:** 10.5005/jp-journals-10071-23150

**Published:** 2019-04

**Authors:** Yuji Takahashi, Tomohiro Sonoo, Hiromu Naraba, Hideki Hashimoto, Kensuke Nakamura

**Affiliations:** 1–5 Department of Emergency and Critical Care Medicine, Hitachi General Hospital, Hitachi, Ibaraki, Japan

**Keywords:** Cardiogenic shock, Critical care, IABP, Sepsis, Septic cardiomyopathy, Intra-arterial balloon pumping for refractory SCM may be an effective method to stabilize circulation status, especially for patients with low heart rates and regular rhythm

## Abstract

**Background and aims:**

Patients with septic cardiomyopathy (SCM) occasionally develop refractory cardiogenic shock, which is difficult to resolve even with the administration of standard dose of catecholamines. Although venoarterial extracorporeal membrane oxygenation (VA-ECMO) has recently been reported with good treatment results, there have been no evidence-based practices. Furthermore, severe SCM may be fatal if the blood pressure cannot be increased. This case series explored whether the application of intra-arterial balloon pumping (IABP) is an effective method for increasing blood pressure in patients with severe SCM.

**Subjects and methods:**

Over a 58-month period, all patients who were admitted in the emergency and critical care center and managed with IABP were investigated. Among these, data sets of patients diagnosed with SCM were evaluated retrospectively.

**Results:**

Ten patients were included in this analysis. Their mean APACHE II and SOFA scores were 26.8±7.9 and 13.9±1.7, respectively. A mean arterial pressure (MAP) increase of more than 30% was achieved in six patients, and a decrease in catecholamine index was observed in five. The effective group consisted of seven patients. The stroke volume increased in 83% of patients who were equipped with pulmonary artery or transpulmonary thermodilution catheter. Low heart rate and regular heart rhythm may be important factors for the effectiveness of IABP for cardiogenic shock caused by refractory SCM.

**Conclusion:**

Intra-arterial balloon pumping may be able to raise MAP in refractory SCM patients even with septic shock by an increase in cardiac output.

**How to cite this article:**

Takahashi Y, Sonoo T, *et al*. Effect of Intra-arterial Balloon Pumping for Refractory Septic Cardiomyopathy: A Case Series. Indian J Crit Care Med 2019;23(4):182–185.

## INTRODUCTION

Some septic patients have impaired cardiac function without ischemia or cardiomyositis. This pathologic state is called septic cardiomyopathy (SCM) and is a septic organ dysfunction. Although the definition of SCM is obscure yet, some recent studies use echocardiography and serum cardiac biomarkers for diagnosis.^1,2^ A few patients with SCM may sometimes exhibit drug-resistant refractory cardiogenic shock.^3–6^

Similar to the standard management of cardiogenic shock, the administration of dobutamine is recommended for refractory SCM after proper fluid resuscitation and noradrenaline administration.^7,8^ Beta stimulants, which are occasionally used for septic shock, may not be as effective in SCM patients due to the downregulation of beta adrenergic receptors caused by inflammatory mediators.^9,10^ Therefore, a few patients with SCM may ultimately exhibit refractory cardiogenic shock since there are currently no evidence-based methods to achieve target blood pressure. In a recent review article, several methods, including IABP and venoarterial extracorporeal membrane oxygenation (VA-ECMO), are introduced as treatments for their refractory shock.^11^ Although VA-ECMO has recently been reported to have 71% survival to discharge^12^ for refractory SCM, its effectiveness has not yet been established. Moreover, IABP for SCM has not been clarified except for a case report.^13^ Generally, IABP supports cardiac function by two effects: (1) systolic-unloading and (2) diastolic-augmentation, where the former may decrease systolic blood pressure while the latter increases diastolic blood pressure. However, these effects have not been validated in septic patients who may have distributive shock. Theoretically, the application of IABP can either increase or decrease mean arterial pressure (MAP). However, in intensive care unit (ICU) settings, we occasionally experience MAP increase and improvement in systemic perfusion status after IABP insertion in septic shock patients with SCM.^13^ We collected cases of SCM patients who were with refractory cardiogenic shock and treated with IABP in our ICU and evaluated the efficacy of IABP for those patients. Our primary interest is the avoidance of acute death from cardiogenic shock, not accomplishing long-term survival.

## SUBJECTS AND METHODS

Our medical and surgical ICU is managed by emergency physicians and accepts all patients from the emergency department. The study term was between July 2013 and April 2018, and all patients managed with IABP in the ICU were investigated. Among these, data sets of patients with sepsis were extracted and examined retrospectively. In this study, SCM patients were defined as follows: satisfied the sepsis-3 criteria,^4^ required the administration of catecholamines, presence of low visual left ventricular ejection fraction (LVEF) confirmed by bedside-echocardiography, and had no clinical evidence for ischemic heart disease or acute cardiomyositis (e.g. changes in electrocardiography or increases in ischemic markers). SCM and stress-induced cardiomyopathy are difficult to clearly distinguish, since SCM is one of stress-induced cardiomyopathy caused by septic inflammatory response. Therefore, we included the patients as SCM if the patient is with low visual LVEF and without troponin elevation. Patients that were previously treated with VA-ECMO when IABP was initiated, diagnosed with acute myocardial infarction, and did not experience cardiogenic shock were excluded. We diagnosed cardiogenic shock based on the Nohria-Stevenson criteria: the simultaneous occurrence of hypoperfusion and conjunction despite adequate preload as well as results obtained from bedside echocardiography, pulmonary artery catheter, and transpulmonary thermodilution method.

The primary outcomes were as follows: increase in MAP of more than 30% and decrease in catecholamine dosage as indicated by the catecholamine index (CAI). CAI was calculated as follows: CAI = dopamine dose + dobutamine dose + (noradrenaline dose + adrenaline dose) × 100 (μg/kg/min). The effective group was defined as the group of patients who achieved at least one of the primary outcomes. In our ICU, the vital sign data are taken every 2 hours, and the change of catecholamine doses is immediately transcribed in the chart. Thus, we decided to measure the outcome by referring the vital signs and catecholamine data which were described right after IABP initiation. The secondary outcomes were the survival rates on the 3rd, 7th, and 28th day after IABP initiation, changes in heart rate and stroke volume, and rate of VA-ECMO escalation. Statistical analysis was not performed due to the small sample size. The outcomes were analyzed by calculating the mean and SD of each parameter. This evaluation was approved by the Research Ethics Committee of Hitachi General Hospital (2013–2048).

## RESULTS

**Fig. 1:**
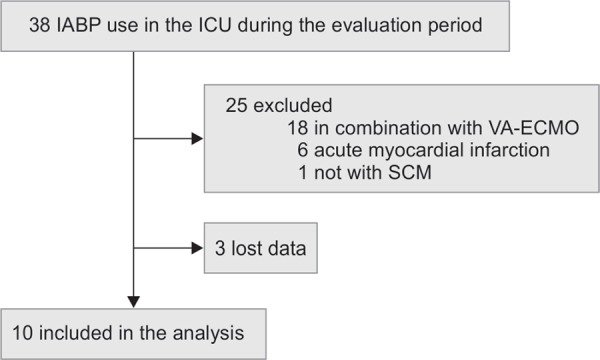
Study population

During the study period, 38 patients were treated with IABP in our ICU. Among these, 28 were excluded: 25 did not meet the inclusion criteria, and three were missing data. Therefore, a total of 10 patients were included (Fig. 1). The base characteristics of these patients are shown in Tables 1 and 2. The mean APACHE II and SOFA scores were 26.8 ± 7.9 and 13.9 ± 1.7, respectively. Cardiac output and stroke volume were recorded in six patients: pulmonary artery catheters were used for five and transpulmonary thermodilution method for one. Coronary angiography was performed in two patients and revealed no stenosis.

For the primary outcomes, a MAP increase of more than 30% was achieved in six patients and a decrease in CAI was observed in five. The effective group was defined as the patient group that achieved at least one of these primary outcomes and consisted of seven patients (Table 3). The noneffective group consisted of the remaining patients who did not achieve at least one of the primary outcomes. Although the overall survival rate on the 28th day was 30%, that on the 3rd and 7th day after IABP initiation were 100% and 70%, respectively, that indicated that the included patients could survive their severe refractory cardiogenic shock with IABP in the acute phase. Stroke volume increased in five of six patients (83%) who were equipped with pulmonary artery or transpulmonary thermodilution catheter (Table 4). We also performed a subgroup analysis on both the effective and noneffective groups. The results showed that the initial heart rates were lower in the effective group. Furthermore, abnormal heart rhythms were observed in two patients (28.6%) in the effective group, whereas all three patients in the noneffective group had abnormal heart rhythms. The three abnormal heart rhythms other than sinus rhythms in the noneffective group consisted of two atrial fibrillations and a series of paroxysmal ventricular contractions accompanied with several ventricular tachycardia. No patients required VA-ECMO in the effective group; however, two eventually required subsequent VA-ECMO due to inadequate cardiac output and multiorgan failure in the noneffective group (Table 5).

**Table 1:** Baseline characteristics of study population

*Case*	*Age/Sex*	*Site of infection*	*CAI*	*Corticosteroid use*	*Preload (CVP, mm Hg)*	*Afterload (MAP, mm Hg)*	*CO (L/min)*	*Lactate (mmol/L) change*	*Urine output (mL/h) change*	*CO (L/min) change*
1	47/male	Unknown	5	No	20	98	1.6	–11.5	15	2.2
2	62/male	Respiratory system	78	No	8	51	NA	1.2	140	NA
3	63/male	Respiratory system	35	No	18	53	1.7	–0.1	5	3.8
4	73/male	Respiratory system	90	Yes	15	80	NA	1.3	–25	NA
5	76/male	Blood stream	30	No	12	90	5.1	0.0	–120	0.8
6	77/female	Respiratory system	300	No	NA	61	NA	–8.1	60	NA
7	79/male	Respiratory system	120	Yes	13	59	3.5	0.5	30	2.5
8	79/male	Urinary tract	5	Yes	14	53	0.8	0.1	–130	1.0
9	83/male	Respiratory system	180	No	24	41	2	–1.6	20	1.9
10	84/male	Urinary tract	100	Yes	13	83	3.5	0.3	20	1.3

**Table 2:** Study population: Highlighting of IABP use (N = 10)

	*IABP use (N=10)*
Age (mean in years)	72.3 ± 11.0
Male, No. (%)	9 (90)
APACHE score	26.8 ± 7.9
SOFA score	13.9 ± 1.7
CAI	94.3 ± 86.2
MAP (mmHg)	67 ± 18
HR (bpm)	97 ± 18
CO (L/min)	2.18 ± 1.00
SV (ml)	25.1 ± 13.3
Lactate (mmol/L)	9.7 ± 6.4
P/F ratio	171 ± 81
*Site of infection*	
Lung (%)	6 (60)
Urinary tract (%)	2 (20)
Blood (%)	1 (10)
Unknown (%)	1 (10)

**Table 3:** Primary outcomes of effective group

		*CAI*		
		*Decrease*	*No change*	*Increase*
MAP	Increase of ≥30%	3	2	1
	Increase between 0–29%	1	0	0
	Decrease	0	2	1

**Table 4:** Primary outcomes of non-effective group

	*IABP use (N = 10)*
*Survival*	
3 day (%)	10 (100)
7 day (%)	7 (70)
28 day (%)	3 (30)
*HR*	
Decrease ≥10% (%)	4 (40)
No change (%)	2 (20)
Increase ≥10% (%)	4 (40)
*SV change*	
No change or decrease (%)	1 (17)
Increase ≥30% (%)	5 (83)
Required subsequent VA-ECMO	2 (20)

**Table 5:** Comparison of effective and non-effective group

	*IABP use (N = 10)*	
	*Effective group (N = 7)*	*Non-effective group (N = 3)*
APACHE score	27.1±8.5	26.0 ± 6.5
SOFA score	13.6±1.6	14.7 ± 1.7
Site of infection		
Lung (%)	5 (71.4)	1 (33.3)
Urinary tract (%)	2 (28.6)	0 (0)
Blood (%)	0 (0)	1 (33.3)
Unknown (%)	0 (0)	1 (33.3)
Original heart rhythm		
Regular sinus rhythm	5 (71.4)	0 (0)
Irregular rhythm	2 (28.6)	3 (100)
Original HR	92 ± 19	107±9
Required subsequent VA-ECMO	0 (0)	2 (67)

## DISCUSSION

To our knowledge, this study is the first case series to report the efficacy of IABP use for cardiogenic shock associated with SCM. In our study, the efficacy of IABP was clarified by MAP increase and CAI decrease in patients whose MAP cannot sustained only by vasoactive agents. Although the mortality rate on 28th day was 30% in our study, the death occurred after all of them had survived severe refractory cardiogenic shock in the acute phase. Furthermore, the implementation of VA-ECMO, an extremely invasive treatment, was avoided in these cases. Therefore, we think IABP is an effective strategy for refractory cardiogenic shock in SCM patients.

Selecting appropriate candidates for IABP is essential since it may also decrease MAP in some patients. Based on our results, patients in the noneffective group had higher heart rates than those in the effective group, and all patients in the noneffective group had irregular heart rhythms. These results indicate that low heart rates and regular rhythm, the synchronicity with IABP, are important factors for IABP to be effective in SCM patients. These factors may help determine SCM patients for whom IABP is effective.

Some treatments have been considered for SCM, such as activated protein C, hydrocortisone, NO synthase inhibitor or methylene blue, however, no specific treatment could show the clinical efficacy.^11,14^ We also suggest acute blood purification, a less invasive but unconfirmed method, to treat refractory shock.^11,13^ Furthermore, a case series reported 71% survival after VA-ECMO for refractory SCM.^12^

Although it has not been previously used as a method of mechanical cardiac support, the results of this study suggest that IABP may be a strategy for refractory SCM. Since refractory SCM is a life-threatening condition and there is no evidence-based practice for it, IABP insertion should be considered prior to initiating VA-ECMO when acute blood purification failed to achieve the target MAP.

There are several limitations to this study. First, this is a case series study which include only 10 patients. It is difficult to generalize the results due to the small sample size. Second, since there is no comparison group for the IABP group, it is so unconvincing to claim the efficacy. A following randomized control trial is required to prove our hypothesis, though it seems difficult because of the rarity of candidates. Third, there is no consensus on the definition of SCM, and, therefore, the accuracy of the diagnosis is unclear. Finally, IABP could not provide improvement of long-term survival, in which most clinicians must be most interested.

## CONCLUSION

Intra-arterial balloon pumping for refractory SCM may be an effective method to stabilize circulation status, especially for patients with low heart rates and regular rhythm.
